# Roles of long noncoding RNAs in colorectal cancer metastasis

**DOI:** 10.18632/oncotarget.16339

**Published:** 2017-03-17

**Authors:** He Li, Si-Qing Ma, Jin Huang, Xiao-Ping Chen, Hong-Hao Zhou

**Affiliations:** ^1^ Department of Clinical Pharmacology, Xiangya Hospital, Central South University, Changsha, P.R. China; ^2^ Institute of Clinical Pharmacology, Central South University, Hunan Key Laboratory of Pharmacogenetics, Changsha, P.R. China; ^3^ Hunan Province Cooperation Innovation Center for Molecular Target New Drug Study, Hengyang, P. R. China

**Keywords:** colorectal cancer, metastasis, lncRNAs, review

## Abstract

Colorectal cancer (CRC) is the 3^rd^ most common malignancies worldwide. Metastasis is responsible for more than 90% CRC patients' death. Long noncoding RNAs (lncRNAs) are an important class of transcribed RNA molecules greater than 200 nucleotides in length. With the development of whole genome sequencing technologies, they have been gained more attention. Accumulating evidences suggest that abnormal expression of lncRNAs in diverse diseases are involved in various biological functions such as proliferation, apoptosis, metastasis and differentiation by acting as epigenetic, splicing, transcriptional or post-transcriptional regulators. Aberrant expression of lncRNAs has also been found in CRC. Besides, recent studies have indicated that lncRNAs play important roles in tumourigenesis and cancer metastasis. They participate in the process of metastasis by activing or inhibiting the metastatic pathways. However, their functions on the development of cancer metastasis are poorly understood. In this review, we highlight the findings of roles for lncRNAs in CRC metastasis and review the metastatic pathways of lncRNAs leading to cancer metastasis in CRC, including escape of apoptosis, epithelial-mesenchymal transition (EMT), angiogenesis and invasion, migration and proliferation. Furthermore, we also discuss the potential clinical application of lncRNAs in CRC as diagnostic markers and therapeutic targets.

## INTRODUCTION

Colorectal cancer (CRC) is the 3^rd^ most commonly diagnosed cancer in males and 2^nd^ in females worldwide. It estimated that 1.4 million new cases and 693,900 deaths occurs in 2012 [1]. Numerous studies suggested that the first stage of metastasis occurs early and that more than 60% of patients have initiated the metastatic process by the time of diagnosis [2]. Based on the degree of metastasis, CRC is divided into localized stage, regional stage and stage with distant metastases. More seriously, compared to CRC patients at localized stage, the 5-year survival rates of patients with distant metastases drop from 90% to 10% [3, 4]. Over the past 3 dedicates, the 5-year survival rates of CRC patients at distant stage has not been improved. Besides, distant metastases lead to approximately 50% death of patients diagnosed with CRC [5]. Obviously, it is necessary to reveal the mechanisms underlying this process. The formation of metastases is a complex and multistep process of the dissemination of tumor cells from the primary tumor microenvironment to various distant organs and colonization of the secondary site [6]. Firstly, tumor cells escape from the primary tumor into the blood or lymphatic system. Secondly, some of them escaping the apoptosis survive and arrest at a secondary site. The third step is extravasation into the distant tissue and survival in the new microenvironment. Finally, they form metastatic colonization by proliferation in the distant location. All these steps are critical for us to understand the biological processes during metastasis. Therefore, angiogenesis, escape of apoptosis, proliferation, invasion and migration are essential for the colonization of metastatic outgrowth [7]. Although numerous genes have been identified as biomarkers of carcinogenesis, the emerging roles of long noncoding RNAs (lncRNAs) in the development of CRC metastasis are largely unknown.

With the advance of whole genome sequencing technology and high-resolution microarray, the massive amount of short RNA or long RNA without protein coding ability was revealed. These non-coding RNAs (ncRNAs) comprise of small interfering RNAs (siRNAs), microRNAs (miRNAs), PIWI-interacting RNAs (piRNAs), small nucleolar RNAs (snoRNAs) and long noncoding RNAs (lncRNAs). Long noncoding RNAs (lncRNAs) are non-coding transcripts of more than 200 nucleotides (nt) in length and majority of them are located in nuclear [8]. LncRNAs are generally transcribed by RNA polymerase II and subsequently are polyadenylated [9]. They have no open reading frame (ORF) resulting in the loss of translation capacity. Similar to protein coding genes, the transcriptional start sites (TSS) of lncRNAs are marked by histone 3 lysine 4 (H3K4me3) and the gene bodies are marked by histone 3 lysine 36 (H3K36me3) throughout. These imply that lncRNAs display epigenetic features [8]. Compared to messenger RNAs (mRNA), most lncRNAs only harbor two exons, resulting that they are shorter than mRNA [9]. Moreover, the expression levels of lncRNAs are significantly lower than mRNA expression and they show developmental and tissue specific expression patterns [10]. Based on the genetic point of view, five different types of lncRNAs are classified, including intergenic lncRNAs, intronic lncRNAs, bidirectional lncRNAs, sense lncRNAs and antisense lncRNAs [9]. These characteristics are essential for us to gain greater insights into the function of lncRNAs.

Recently, lncRNAs have caught more attention and accumulating studies suggested that lncRNAs play critical roles in tumourigenesis and cancer progression. They function as oncogenes and tumor suppressors in diverse biological processes, such as imprinting, epigenetic regulation, apoptosis, cell cycle, transcriptional and translational regulation, splicing, cell development and differentiation [11]. Besides, numerous studies have demonstrated that The metastatic pathways were highly associated with the aberrant expression of lncRNAs and lncRNAs played important roles in the development of metastases by activing or inhibiting the metastatic pathway in different cancers, such as hepatocellular carcinoma, gastric cancer and non-small cell lung cancer [12–14]. Due to the tumor or tissue specific expression patterns of lncRNAs, there is a critical need for amount of researches on the association between the dysregulation of lncRNAs and the metastatic pathways in CRC. However, the number of data is still limited. With the development of the therapeutic approaches based on lncRNAs, it is necessary to summarize the role of lncRNAs in the development of metastasis, especially in CRC.

In this review, we highlight the findings of roles for lncRNAs in CRC metastasis and review the association of lncRNAs and metastatic pathways in CRC, including escape of apoptosis, epithelia-mesenchymal transition (EMT), angiogenesis and proliferation, invasion and migration [7]. Furthermore, we also discuss the potential clinical application of lncRNAs in CRC.

## LNCRNAS INVOLVED IN CRC METASTASIS

Recently, accumulating evidences have uncovered that lncRNAs directly regulate the metastatic pathways in CRC [15, 16]. By using Human lncRNA Assay, Han J *et. al* compared lncRNA expression profiles between metastatic lymph node (MLN), normal lymph node (NLN) and tumor tissues from three CRC patients. Five lncRNAs were down-regulated and fourteen lncRNAs were up-regulated in the MLN group compared with the NLN group and tumor tissue group. Besides, four gradually up-regulated lncRNAs and sixty-six down-regulated lncRNAs were identified from tumor tissue to MLN and NLN, respectively. All these lncRNAs were supposed to play important roles in the lymph node metastasis (LNM) of CRC [17]. Recently, three pairs of tumor tissues and MLNs were used to perform in another microarray analysis. A total of 390 aberrant expressed lncRNAs were observed in the tumor tissues compared with MLNs. Especially, ENST00000430471 exhibited the lowest expression in tumor tissues compared with MLNs. Further studies suggested that it promoted cell proliferation, invasion, migration and S-phase arrest and inhibited cell apoptosis [18]. To identified the role of lncRNAs in the progress of colorectal liver metastasis (CLM), Ye LC *et. al* profiled the lncRNAs expression in CRC tissues with synchronous, metachronous and non-liver metastasis. Three novel lncRNAs of forty differentially expressed lncRNAs in CLM tissues, were verified to be involved in CLM [19]. Besides, a genome-wide analysis comparing lncRNAs expression profiles between CRC tissues with or without liver metastasis identified 2636 differentially expressed lncRNAs. Six lncRNAs (POU6F2-AS1, RAB6C-AS1, DDP10-AS1, HOXA11-AS, LINC00944 and FEZF1-AS1) were verified to participate in the process of liver metastasis in CRC though further validation [20]. Interestingly, FEZF1-AS1 was subsequently reported to enhance CRC cells proliferation, invasion and migration partly through FEZF1 induction [21]. Recently, genome-wide lncRNA expression patterns were assessed by microarray analysis in metastatic lymph nodes and its paired normal lymph nodes of CRC patients. A new lncRNA named GAPLINC was found. It was demonstrated to be associated with tumor growth, tumor stage, node stage and overall survival of CRC. *In vivo* and *vitro* assays, it significantly promoted cell proliferation and invasion by interacting with PTB-associated splicing factor (PSF) and non-POU-domain-containing octamer-binding (NONO) [22]. All their results supposed that lncRNAs may play crucial roles in the metastasis of CRC.

Although the use of microarray makes to identified accumulate of lncRNAs, which are significantly associated with the process of CRC metastasis. Microarray analysis on colorectal tumor at multi-treatment center, of different subtypes and with large sample sizes were blank. Furthermore, to fully utilize the microarray data and better understand the intrinsic mechanisms, the specific signaling of these screened lncRNAs involved in influencing CRC metastasis should be investigated. Therefore, more studies *in vivo* and *vitro* are critical needed to continue.

## LNCRNAS AND METASTATIC PATHWAYS IN CRC

Multivariate evidences have demonstrated that aberrant expression of lncRNAs plays important roles in the metastatic process both *in vitro* and vivo in CRC (Table 1). Many metastatic pathway including escape of apoptosis, EMT, angiogenesis and invasion, migration and proliferation are crucial for the formation of metastases. Therefore, we discuss and describe the association of lncRNAs and these metastatic pathways in CRC in detail below.

**Table 1:** lncRNAs involved in metastasis CRC

lncRNA	Chromosomal location	Gene type	Putative functions related to metastatic prognosis	Signaling pathways	Ref.
CLMAT3	Chr14	Oncogene	Liver metastasis	G0/G1 cell-cycle arrest	[19]
FEZF1-AS1	7q31.32	Oncogene	Proliferation, migration, invasion and metastasis		[20, 21]
GAPLINC	Chr18	Oncogene	LNM, proliferation and invasion	GAPLINC/PSF/NONO	[22, 105]
PVT-1	8q24	Oncogene	LNM, Proliferation, invasion and apoptosis		[25]
DQ786243	Chr1	Oncogene	Cell cycle, apoptosis, metastasis proliferation and invasion,	G2/M cell-cycle arrest	[26]
BANCR	Chr9	Oncogene	Proliferation, migration, EMT, cell cycle, and apoptosis	EMT signaling pathway	[28, 53, 167]
HOTTIP	7q15.2	Oncogene	Distance metastasis, proliferation and apoptosis	G0/G1 cell-cycle arrest	[35, 37]
LincRNA-p21	6p21.2	Tumor suppressor	Vascular invasion, proliferation and cell cycle	Wnt/β-catenin signaling pathway	[38–40, 42, 116, 117]
ZFAS1	20q13.13	Oncogene	Proliferation, apoptosis and cell cycle	p53-dependent cell cycle	[43]
Loc554202	9p21.3	Tumor suppressor	Proliferation, tumorigenesis and apoptosis	caspase cleavage cascades	[45]
UCA1	19p13.12	Oncogene	Proliferation, metastasis, cell cycle and apoptosis,,		[46, 104]
PRNCR1	8q24.21	Oncogene	Proliferation and cell cycle	G0/G1 cell-cycle arrest	[47, 168]
HOTAIR	12q13.13	Oncogene	Proliferation, invasion, EMT, LNM and Lung metastasis	EMT signaling pathway	[52]
TUG1	22q12.2	Oncogene	EMT, invasion and migration	EMT signaling pathway	[54]
LncRNA-ATB	Chr14	Oncogene	LNM, EMT, tumorigenesis and invasion	EMT signaling pathway	[56–58]
H19	11p15.5	Oncogene	Proliferation, EMT and cell cycle	EMT signaling pathway β-catenin pathway	[64, 106–108]
CTD903	14q11.2	Oncogene	Invasion and migration and EMT	EMT signaling pathway Wnt/β-catenin pathway	[65]
GHET1	7q36.1	Oncogene	Proliferation, invasion, migration and EMT	EMT signaling pathway	[66]
CASC2	10q26.11	Tumor suppressor	Proliferation and cell cycle	G0/G1 cell-cycle arrest	[109]
LOC285194	3q13.31	Tumor suppressor	Distant metastasis and proliferation		[110, 111]
FER1L4	20q11.22	Tumor suppressor	LNM, cell cycle, proliferation, invasion, and migration		[113]
CCAL	Chr3	Oncogene	Proliferation, invasion, migration, apoptosis, cell cycle and LNM		[118]
CCAT1	8q24.21	Oncogene	LNM, proliferation and invasion		[120]
CASC11	8q24	Oncogene	Lymph metastasis and proliferation	Wnt/β-catenin pathway	[121]
CCAT2	8q24.21	Oncogene	Cell growth and metastasis		[122]
PCAT-1	8q24.21	Oncogene	Distant metastasis		[123]
MALAT1	11q13.1	Oncogene	Proliferation, migration, invasion and metastasis	PI3K/Akt pathway Wnt/β-catenin pathway	[124–127]
TINCR	19q13.3	Tumor suppressor	Proliferation and metastasis	Wnt/β-catenin pathway	[128]
ncRAN	17q25.1	Tumor suppressor	Migration and invasion		[129]
RP11-462C24.1	4q25	Tumor suppressor	Distant metastasis		[130]
91H	7F5; 7.87.97cM	Oncogene	Distant metastasis, migration, invasion and proliferation		[131]
MEG3	14q.32	Tumor suppressor	Proliferation, invasion and metastasis		[132, 133]
lncRNA-LET	15q24.1	Tumor suppressor	Invasion and metastasis		[134]
FTX	Xq13.2	Oncogene	Lymph vascular invasion, proliferation, invasion and migration		[135]
NEAT1	11q13.1	Oncogene	Proliferation, invasion and metastasis	Akt signaling pathway	[136, 137]
DANCR	4q12	Oncogene	LNM		[138]
AFAP1-AS1	4p16.1	Tumor suppressor	Distant metastasis, proliferation and cell cycle		[139]
GAS5	1q25.1	Tumor suppressor	Distant metastasis, proliferation and cell cycle		[140]
ANRIL	9q21.3	Oncogene	Proliferation, invasion and migration		[141]
ncRuPAR	5q13.3	Tumor suppressor	LNM and distant metastasis		[142]
CRNDE-h	Chr16	Oncogene	LNM and distant metastasis		[143]

### LncRNAs and escape of apoptosis

**Figure 1:**
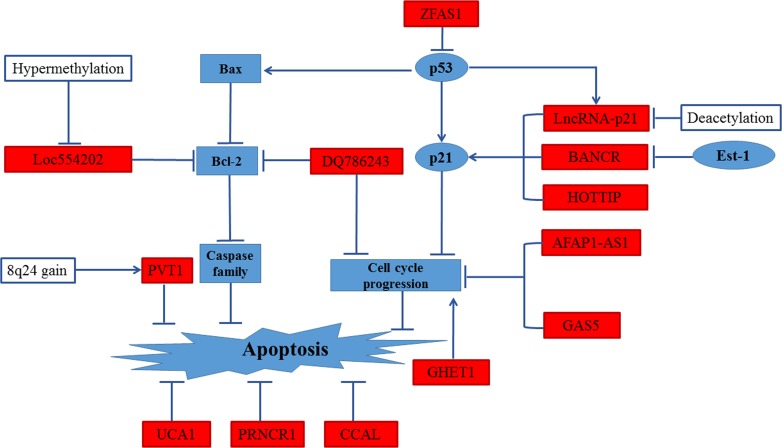
Regulation of apoptosis in CRC by lncRNAs UCA1, PRNCR1 and CCAL could regulate the apoptosis pathway in CRC. High expression levels of PVT-1 response to 8q24 copy-number gain inhibited apoptosis pathway. Low expression levels of Loc554202 inhibited apoptosis pathway by down-regulation of Bcl-2. DQ786243 down-regulated Bcl-2 expression and led cell cycle arrest, leading to repressing apoptosis pathway. GHE1, GAS5 and AFAP1-AS1 influenced apoptosis by regulating cell cycle progression. HOTTIP modified apoptosis pathway and cell cycle progression by inducing expression of p21. BANCR, regulated by Est-1 mildly effected proliferation by promoting G1 arrest and causing p21 mediated- apoptosis. lincRNA-p21 regulates the G1/S the checkpoint and proliferation by promoting p53-dependent transcription of p21. ZFAS1 may influence cell cycle progress and inhibit apoptosis *via* destabilization of p53

The evasion of apoptosis and shear stress in the vasculature leading to arrest at a secondary site is an important step of metastatic process. Tumor cells with the phenotype of anti-apoptosis are more likely to metastasize [23]. Several researches have described the regulation of lncRNAs on cell cycle arrest and apoptosis [24]. The functional relationship of various lncRNAs in the escape of apoptotic cell death are reviewed in Figure 1.

PVT1, which maps to 8q24, is a novel promising biomarker in different solid cancers including CRC [25]. In CRC, high expression levels of PVT-1 in response to 8q24 copy-number gain showed greater lymph node metastasis (LNM). By gene expression microarray assays on CRC cell lines transfected with PVT-1 siRNA and NC group, it was demonstrated that apoptosis was induced by knockdown of PVT-1 in CRC cells [25]. More recently, another lncRNA DQ786243 was found to be differentially expressed between CRC tissues and adjacent normal tissues. *In vitro*, knockdown of DQ786243 was proved to inhibit cell proliferation, invasion and migration. In addition, DQ786243 is suggested to be involved in apoptosis and cell cycle progression [26].

Activation of the p53 signal pathway was proposed to play critical roles in both cell cycle arrest and apoptosis. p21, a key downstream effector of p53, was activated through the p53 dependent or independent pathway to inhibit cell proliferation by inducing G0/G1 arrest and apoptosis [27]. By examining BANCR levels in a cohort of 38 CRC patients, Shi *et. al* demonstrated that BANCR was low expressed in CRC and might be a promising biomarker for prognosis in CRC. In SW480 and HCT116 cells, BANCR mildly effected proliferation by promoting G1 arrest and causing p21 mediated- apoptosis [28]. As an oncogene, HOTTIP promotes cell proliferation, migration and inhibits cell apoptosis in different cancers [29–36]. In CRC, increased HOTTIP expression was supposed to be an unfavorable and independent prognostic factor for its association with clinical stage and distant metastasis [35]. *In vitro* assay suggested that HOTTIP inhibited G0/G1 arrest and promoted CRC cell growth partly by silencing of p21 expression [37]. Compared to healthy control, lincRNA-p21 was not only decreased in CRC tissues but also in plasma of CRC patients [38, 39]. Besides, numerous evidences revealed that lincRNA-p21 activated p53-mediated apoptosis pathway [40, 41]. To further explore the effects of lincRNA-p21 on the control of expression of p53 target genes, a conditional knockout mouse model was generated. Their results showed that lincRNA-p21 regulates the G1/S the checkpoint and proliferation by promoting p53-dependent transcription of p21 [42]. Recent studies demonstrated that ZFAS1 was identified as an oncogene in CRC. It may influence cell cycle progress and inhibit apoptosis *via* destabilization of p53 and through interaction with CDK1/cyclin B1 complex [43].

As we all know, caspase cleavage cascades act as the dominant regulator in the death signaling [44]. Due to the hypermethylation, Loc554202 was significantly decreased in human CRC tissues and CRC cell lines compared to controls. After transfection with pCDNA-Loc554202, the number of cells in the S-phase was obviously reduced and the proportions of apoptotic cells was significantly increased. Further studies indicated that activation of specific caspase cleavage cascades was partly responsible for Loc554202-induced CRC cell apoptosis [45]. Besides, UCA1 and PRNCR1 were both reported to inhibit cell cycle and apoptosis [46, 47]. However, UCA1 was pointed out to contribute to apoptosis by suppression of p53 expression in breast cancer cells and cardiomyocytes [48, 49], the exact mechanisms in CRC are still unclear.

### LncRNAs and EMT

**Figure 2:**
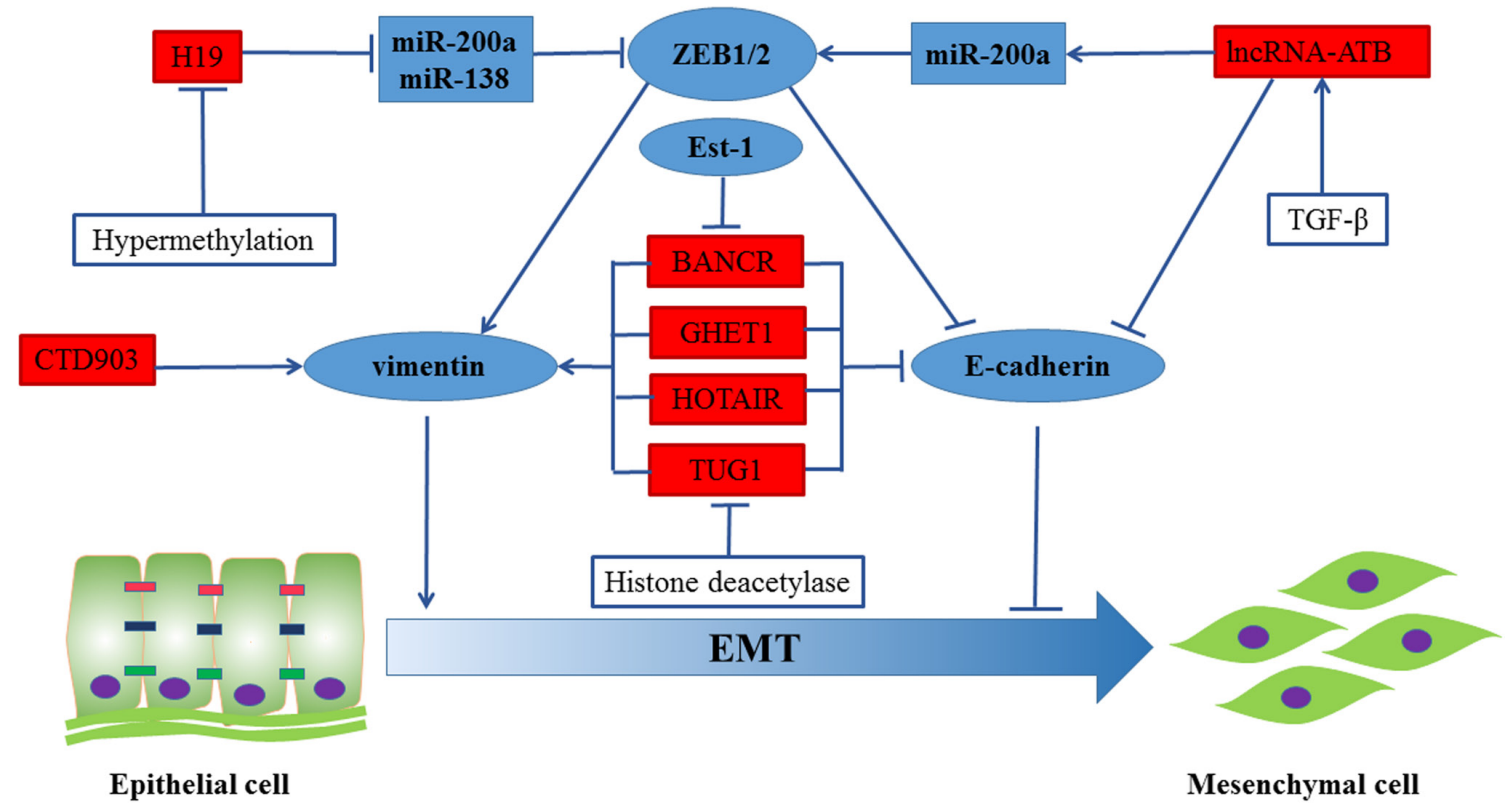
Regulation of EMT in CRC by lncRNAs BANCR, GHET1, HOTAIR and TUG1 induced EMT phenotypes by repressing the expression of vimentin and promoting the expression of E-cadherin. The expression of long non-coding RNA-activated by TGF-β (lncRNA-ATB) mediated epithelial markers (E-cadherin, ZO-1) repression and increased the expression of mesenchymal markers ZEB1 and N-cadherin through sequestering miR-200a. Besides, H19 significantly promoted EMT progression by functioning as a ceRNA for miR-138 and miR-200a. Moreover, CTD903 induced EMT-like phenotypes.

EMT is one of the underlying driving forces for primary tumor cells to acquire a migratory capacity and metastasis [50]. In addition, EMT property is also linked with a stem cell-like phenotype in invasive, de-differentiated cancer cells by analyzing gene expression patterns in CRC and their corresponding liver metastases [51]. Large numbers of lncRNAs have been reported to regulate EMT progress. Therefore, we summarized the regulation of EMT by lncRNAs in Figure 2.

Overexpression of zinc-finger-enhancer binding protein 1/2 (ZEB1/2), promotion of vimentin transcription and repression of E-cadherin transcription are accompanied by the activation of EMT. Recent study found out that HOTAIR, highly expressed in CRC stem cells, regulated the expression of EMT- associated molecules expression, including E-cadherin and vimentin/N-cadherin [52]. High-BANCR expression group was reported to be associated with more advanced LNM than the low- BANCR expression group. To further understand the internal mechanism, E-cadherin and vimentin were detected in HCT116 cells and Caco-2 cells. The results suggested that BANCR induced EMT phenotypes by repressing the expression of vimentin and promoting the expression of E-cadherin [53]. *In vitro* study pointed out that TUG1 played a critical role in CRC metastasis by activating EMT progress [54, 55]. The expression of long non-coding RNA-activated by TGF-β (lncRNA-ATB) was higher in metastatic cancer tissues [56]. Besides, lncRNA-ATB mediated epithelial markers (E-cadherin, ZO-1) repression and increased the expression of mesenchymal markers ZEB1 and N-cadherin through sequestering miR-200a [57, 58]. H19 was identified as a novel regulator of EMT in CRC. Multiple studies demonstrated that H19 may act as a competing endogenous RNA (ceRNA) [59–63]. In Liang's study, it was reported that H19 was highly expressed and significantly promoted EMT progression. It mainly functioned as a ceRNA for miR-138 and miR-200a. to repress vimentin and ZEB2 expression, which were their endogenous targets and core mesenchymal markers [64]. Recently, the correlation of CTD903 expression and lymphatic and distant metastasis was observed in 115 pairs of CRC tissues. After treating with CTD903 siRNA, RKO and SW480 CRC cells exhibited the typical mesenchymal cell morphology, indicating that reduction of CTD903 induced EMT-like phenotypes. Further studies revealed that CTD903 repressed Wnt/β-catenin signal pathway and down-regulated the expression of Twist and Snail, whereas it had no effect on the expression of E-cadherin/N-cadherin and ZEB1 [65]. In addition, GHET1was also demonstrated to be involved in EMT prognosis in CRC. But, how it functions in CRC are still not reported [66]. Recently, SPRY4-IL1 and PANDAR both were proved to promote CRC metastasis *via* EMT pathway [67–69]. Besides, LINC01133 and SLC25A25-AS1 were tumor suppressors in CRC. Low levels of LINC01133 and SLC25A25-AS1 were suggested to promote EMT in CRC [70, 71]. Downregulation of SCL25A25-AS1 promoted EMT process dependent on Erk and p38 signaling. However, EMT process was regulated by LINC01133 dependent on the presence of SRSF6.

### LncRNAs and angiogenesis

It has been demonstrated that tumor progresses are the development of tumor cells from prevascular phase to vascular phase. After the prevascular phage, capillaries were newly formed surrounding the tumor stroma, passed nutrients to it and allow tumor cells to enter into the circulation. Thus, compared to tumors in prevascular phase, vascularized tumors induced angiogenesis, are large in size and have a propensity to metastasize [72]. Angiogenesis is a complex process of new blood vessels formation. The first step is degradation of the extracellular matrix (ECM) and sprouting of the endothelial cells toward the gradient of vascular endothelial growth factor (VEGF). The next step is differentiation of the endothelial cells into tip, stalk and tube cells. Subsequently, it is the tube formation and maturation [73]. More and more experimental and clinical evidences suggest that angiogenesis is a hallmark of tumor metastasis and growth. It is significantly associated with advanced tumor growth and distant metastases in CRC [74]. Therefore, many anti-angiogenic agents were developed, including the VEGFA-targeted antibody bevacizumab [75]. Bevacizumab combined with capecitabine or irinotecan, fluorouracil and leucovorin chemotherapy can extend progression-free and overall-free survival in metastatic colorectal cancer (mCRC) [76–78].

**Figure 3:**
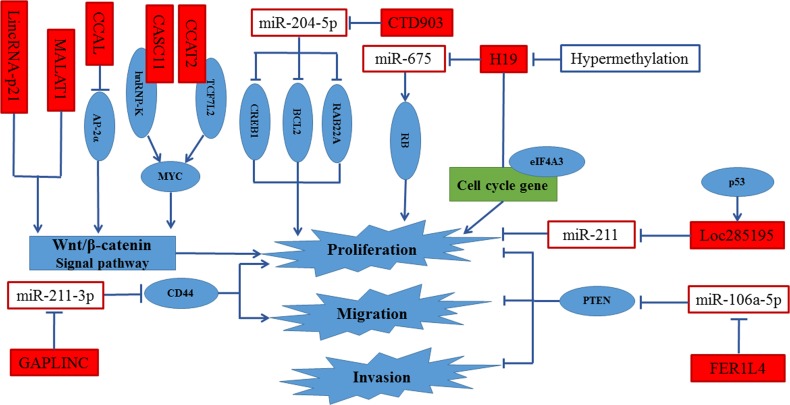
Regulation of proliferation, invasion and migration in CRC by lncRNAs MALAT1 and lncRNA-p21 were supposed to promote cell proliferation, invasion and migration by activating Wnt/β-catenin signal pathway. CCAL enhanced cell proliferation by activated AP-2α-mediated Wnt/β-catenin signaling. CASC11 and CCAT2 activated Wnt/β-catenin signal pathway by directly targeting hnRNP-K and TCF7L2, respectively. CTD903, Loc285195 and H19 promoted proliferation through sequestering miR-204-5p, miR-211 and miR-675, respectively. Besides, the binding of eIF4A3 to H19 decreased the recruiting of eIF4A3 to the cell-cycle gene mRNA, resulting in the promotion of cell proliferation.

Accumulating evidences proved that lncRNAs played critical roles in angiogenesis [79]. A lncRNA termed MVIH (lncRNAs associated with microvascular invasion in hepatocellular carinoma) was firstly reported to promote tumor growth and metastasis by activating angiogenesis. Using RNA pull-down (RIP) assays and enzyme-linked immunosorbent assays (ELISA) respectively, it was suggested that lncRNA MVIH activated angiogenesis *via* inhibiting the secretion of PGK1 (phosphoglycerate kinase 1), which is critical for angiogenesis [80]. Subsequently, MALAT1 was also proved to promote angiogenesis *in vitro* and vivo. The human umbilical vein endothelial cells (HUVECs) transfected with MALAT1 siRNA or GapmeRs directed against MALAT1 showed a significantly higher number of sprouts and more cell migration. Moreover, MALAT1 was observed to activate angiogenesis by regulating vessel density, vessel extension and blood flow recovery in MALAT1^−/−^ mouse model [81, 82]. Recently, Microarray data of gene expression profiles suggested that MALAT1 promoted the secretion of fibroblast growth factor 2 (FGF2) in neuroblastoma cells under hypoxic conditions. It indicated that MALAT1 play a critical role in angiogenesis [83]. In osteoarthritis, MEG3 is found to be inversely associated with VEGF levels, which is involved in angiogenesis [84]. Therefore, we guess that MEG3 may regulate angiogenesis by influence the expression of VEGF. Moreover, HULC was proved to promote angiogenesis in gliomas and liver cancer [85, 86]. In glioma patients' tissues, HULC was found positively associated with VEGF and microvessel density. Furthermore, the vitro assay results in U87MG and U251 cells showed that HULC silencing suppressed angiogenesis by inhibiting cell proliferation and invasion *via* PI3K/mTOR signaling pathway [85]. Besides, it was suggested to induce angiogenesis by upregulating sphingosine kinase 1 (SPHK1) [86]. Recently, accumulating lncRNAs, including H19, lincRNA-p21, TUG1 and HOTAIR, was proved to be involved in angiogenesis in different cancers [87–90]. Interestingly, all these lncRNAs have been proved to be involved in CRC metastasis. Therefore, they may affect CRC metastasis partly by regulating angiogenesis. However, there is no report about the functions of lncRNAs on angiogenesis in CRC. More importantly, by using next-generation ribonucleic acid sequencing and microarray assay, Fiedler J *et. al* found that LINC00313 and MIR503HG were significantly associated with angiogenesis. They also verified their potential clinical importance in an *ex vivo* model of human induced pluripotent stem cell-based engineered heart tissue [91]. These evidences suggested that lncRNA-based therapeutic strategies hold great promise to modulate tissue vascularization and be used to CRC treatment.

### LncRNAs and proliferation, invasion and migration

Numerous studies show that microRNAs (miRNAs) play important roles in CRC metastasis. They are involved in various metastatic pathways of CRC [7]. LncRNAs, identified as the one kind of naturally competing endogenous RNAs (ceRNAs), inhibit the repression of miRNAs targets *via* competing for binding of the cellular miRNAs [92, 93]. Therefore, accumulating lncRNAs were found to serve as microRNAs sponges to regulate proliferation, invasion and migration in CRC. Urothelial carcinoma associated 1 (UCA1) was suggested to serve as a biomarker of several solid cancers including bladder cancer, breast cancer, gastric cancer and CRC. It was reported that UCA1 was a transcriptional target of C/EBPα, HIF-1α and Ets-2 [94–97] . It was demonstrated to regulate different pathways, including PI3K, AKT, KLF4-KRT6/13, mTOR-STAT3 and p27Kip1/CDK2 signaling [98–101]. Besides, it also was identified as a ceRNA [102, 103]. In CRC cells, UCA1 was proved to enhance cell proliferation by inhibiting the function of miR-204-5p *via* partly controlling its target genes [104]. By analyzing the expression of 45 pairs CRC tissue samples, a newly identified cancer-related lncRNA, lncRNA-uc002kmd.1(GAPLINC) was considered to be a key regulator of CRC. Their results also proved that lncRNA-uc002kmd.1 formed a molecular decoy for miR-211-3p, which targets CD44 for degradation. The lncRNA-uc002kmd.1-drived CD44-dependent cell growth by competing for miR-211-3p is respond for the cell proliferation and tumor growth *in vitro* and vivo [105]. LncRNAs are also identified as the precursor of miRNAs [106]. In CRC, H19 was shown to be the precursor of miR-675. H19 was associated with miR-675 expression in CRC cell lines and CRC tissues. Subsequently, the tumor suppressor retinoblastoma (RB) was proved to be a direct target of miR-675 by dual-luciferase reporter gene assay. *In vitro* assay, H19 promoted CRC cell proliferation by H19/miR-675/RB pathway [107].Besides, the binding of eIF4A3 to H19 decreased the recruiting of eIF4A3 to the cell-cycle gene mRNA, resulting in the promotion of cell proliferation. Interestingly, the results showed that H19 promoted cell proliferation by only regulating cell cycle progression but not apoptosis. [108]. Recently, evidences have suggested that Cancer Susceptibility Candidate 2 (CASC2) serves as an oncogene in CRC. Overexpression of CASC2 inhibits cell proliferation by extending G0/G1-S phase transition. Further studies indicated that CASC2, as a ceRNA by sponging miR-18a, activated the STAT3 signal pathway by modulating the level of the miR-18a targets [109]. Moreover, low expression of LOC285194 was correlated with larger tumor size and more distant metastasis [110]. *In vitro* and vivo assays, LOC285195, a transcriptional target of p53 was suggested to inhibit cell proliferation and tumor growth by repressing the expression of miR-211 [111]. FER1L4 is involved in various cancers including gastric cancer and colon cancer [112, 113]. In CRC, FER1L4 expression levels exhibited a negative associated with LNM, vascular invasion and depth of tumor invasion. Notably, 86.1% lymph node metastatic tissues showed lower expression of FER1L4 compared with primary cancer tissues. By gain-of- function assays, FER1L4 was revealed to inhibit cell proliferation, migration and invasion.

Wnt/β-catenin signal pathway plays a critical role in colorectal carcinogenesis. It controls not only EMT but also cell proliferation, invasion and migration in CRC [114]. As a tumor suppressor, LincRNA-p21 has been reported to inhibit the translation of β-catenin in Hela cells [115]. By detecting the expression of lincRNA-p21, β-catenin and Wnt/β-catenin target genes in 30 CRC tissues and its adjacent tissues, an inverse correlation between lincRNA-p21 and activity of β-catenin was found. Further studies demonstrated that lincRNA-p21 significantly repressed the Wnt/β-catenin signal pathway [116]. Subsequently, the results were verified in stem-like CRC tissues and cells [117]. By globally analyzing the protein-coding RNA and lncRNA expression profiles of normal colorectal tissues, colorectal adenoma and CRC tissues, a non-annotated lncRNA, CCAL, was emerged as oncogene. Using qRT-PCR analysis in 252 CRC tumor and paired non-tumor tissues and CRC cells, Ma *et al* revealed that CCAL overexpression was a frequent event no matter in CRC tissues and cells. *In vitro* and vivo assay, CCAL was suggested to be involved in several biological functions, including cell proliferation, invasion, migration, apoptosis and tumorigenesis. Further studies demonstrated that CCAL enhanced cell proliferation by activated AP-2α-mediated Wnt/β-catenin signaling [118]. Numerous studies have demonstrated that Multiple genes, located at 8q24, are often amplified and involved in metastasis in CRC [119]. Until now, six lncRNAs (PVT-1, PCAT-1, PRNCR1, CASC11, CCAT1 and CCAT2), all mapping to 8q24, were reported to overexpress in CRC [25, 47, 120–123]. By RNA-binding protein immunoprecipitation (RIP) experiments and western blot assays, CASC11 and CCAT2 activated Wnt/β-catenin signal pathway by directly targeting hnRNP-K and TCF7L2, respectively [121, 122]. Besides, MALAT1 was suggested to be a prognostic biomarker in in stage II/III CRC patients [124]. In LoVo cells, MALAT1 was supposed to promote cell proliferation, invasion and migration by increasing the nuclear localization of β-catenin, resulting in activating Wnt/β-catenin signal pathway [125]. More than Wnt/β-catenin signal pathway activation, it is reported that MALAT1 promotes cell proliferation, invasion and migration not only *via* PRKA kinase anchor protein 9 but also through binding to SFPQ and releasing oncogene PTBP2 from SFPQ/PTBP2 complex [126, 127]. Recently, TINCR was proved to correlate with CRC proliferation and metastasis *in vivo* and *vitro*. The potential mechanisms was that loss of TINCR enhanced hydrolysis of EpCAM, subsequently, activated Wnt/β-catenin signal pathway [128]. Besides, several other lncRNAs were reported to participate in the prognosis of cell proliferation, invasion and migration in CRC [129–143]. However, the potential mechanisms are still unclear.

## FUTURE PERSPECTIVES AND CLINICAL APPLICATION

As discussed in above, lncRNAs are involved in different biological progressions in CRC. Moreover, similar to other nucleic acids, lncRNAs can also be detected in peripheral blood, such as serum, plasma, and peripheral blood mononuclear cells. Therefore, it likely that circulating lncRNAs may be new non-invasive molecular markers for tumor diagnosis [144, 145]. We list some lncRNAs with potential prognostic value for CRC diagnosis and therapy in Table 2. Recent studies showed that the combination of two exosomal mRNAs, KRTAP5-4 and MAGEA3 and one exosomal lncRNA, BCAR4 in serum could be potential candidates to detected CRC [146]. Moreover, by detecting 13 lncRNAs in serum samples from 71 CRC patients and 70 healthy individuals, it was suggested that a three- lncRNA signature (LOC285194, rp11-462C24.1 and Nbla12061) show potential as a diagnostic marker for CRC. More importantly, it showed much higher diagnostic ability than conventional blood biomarkers 0.793 (95% CI: 0.709 to 0.861), such as carcinoembryonic antigen (CEA), carbohydrate antigen 199 (CA199), carbohydrate antigen 125 (CA125) and carbohydrate antigen 724 (CA724) [147]. However, lncRNA-based tumor diagnostics has not been developed for use yet.

**Table 2:** LncRNA with potential biomarkers for CRC diagnosis and prognosis

LncRNAs	Origin	n	AUC	95%Cl	Sensitivity(%)	specificity(%)	Ref.
BANCR, NR_026817, NR_029373 and NR_034119	Serum	120	0.881	0.833.919	89.17	75.83	[169]
PRNCR1	Tissue	63	0.799		80.4	70.0	[47]
CCAT1&HOTAIR	Plasma	32	0.954	0.903-1.000	84.3	80.2	[38]
LOC285194, RP11-462C24.1 and Nbla12061	Serum	70	0.793	0.709-0.861	68.33	86.89	[170]
MEG3 (diagnosed for CLM)	Tissue	51	0.62	0.48-0.74			[171]
GAS5 (diagnosed for CLM)	Tissue	51	0.65	0.51-0.77			[171]
H19(diagnosed for CLM)	Tissue	51	0.56	0.43-0.69			[171]
Yiya(diagnosed for CLM)	Tissue	51	0.70	0.56-0.81			[171]
NEAT1_1	Blood	30	0.732	0.724-0.842	56.7	83.3	[172]
NEAT1_2	Blood	30	0.845	0.816-0.914	86.6	83.3	[172]
CRNDE-h	Plasma	15	0.888		87	93	[173]
BCAR4 and 2 mRNA	Exsome	30	0.936	0.840-0.983			[146]

Because of the critical roles of lncRNAs in cancer, modulation of lncRNAs expression seems to have vast potential in developing lncRNA-based cancer therapy. Based on viral and non-viral vectors, several efficient delivery systems to alter expression of lncRNA have been developed. Due to the serious side effects, non-viral inhibition of lncRNA is more practical in clinic. Non-viral inhibition of lncRNA is performed either with small molecule inhibitors or oligonucleotide -based therapeutics (antisense oligonucleotides and RNAi mediated gene silencing) [148]. Small molecule inhibitors are used to block the binding sites or change the secondary structure of the lncRNA, resulting in disrupting the interactions between lncRNAs with proteins or nucleic acids. However, there is still a huge challenge and more efforts are needed [149].

Currently, RNA interference (RNAi) and antisense oligonucleotides (ASOs) are another two applicable oligonucleotide based therapeutic approaches. RNAi is a specific, safe and cost-effective way to mimic the natural way of gene silencing by using synthetic siRNAs [150]. It has demonstrated that exogenous siRNAs are able to deplete lncRNA molecules within cytoplasm and nucleus [151]. Surprisingly, more evidences suggested that knockdown of some lncRNAs including HOTAIR and MALAT1 by siRNA induced significant anticancer effect *in vivo* and *vitro* [152, 153]. Until now, several formulation of siRNAs are undergoing different phase of clinical trials. However, all the siRNAs are targeting mRNAs not lncRNAs. Moreover, how to achieve successful siRNA delivery in order to make it stable in circulation, reaching the target tissue accurately, entering the cancer cells and load into the RISC complex successfully is still the main obstacle. Even through several strategies such as modifications of the siRNA molecules, the use of nanoparticle and lipid-based delivery tools can partly solve these issues, Obviously, the therapy using siRNAs targeting critical lncRNAs is still in the infancy stage and needs a long way to go. ASOs are short (13-25 nt) single stranded oligonucleotides complementary to the target RNA. They are used to down-regulate lncRNA in the nucleus by blocking lncRNAs and inducing lncRNAs degradation by nucleases [154]. Compared with RNAi, ASOs can not only knockdown the targeting lncRNAs effectively *in vitro* and vivo, but also show superiority over siRNAs in safety. Notoriously, lncRNAs can serve as endogenous microRNA sponge to reduce its activity. Similarly, evidences showed that microRNAs may naturally regulate lncRNAs expression vice versa [155].

Except RNAi, ASOs and microRNAs, hammerhead ribozyme (HanRz) and aptamer also show good inhibitory effect. However, some lncRNAs may be virtually undruggable, because of their low transcript abundance. Meanwhile, all these pharmaceutical technologies are aimed at the oncogenic lncRNAs. The important roles of other lncRNAs served as tumor suppressors in cancer should not be overlooked.

## CONCLUSIONS

Evidence is accumulating that lncRNAs play critical roles in cancer progression and metastasis. In this review, we summarized the dysregulated lncRNAs associated with CRC metastasis. By microarray analysis, lncRNA expression profiles in primary tumors and metastatic tissues including MLN tissues and CLM tissues were compared [17–20, 22]. All these results supposed that lncRNAs were associated with the metastatic phenotype in CRC patients. However, the data are limited and microarray analysis on colorectal tumor at multi-treatment center, of different subtypes and with large sample size should be performed. Besides, we described the association of lncRNAs and metastatic pathways in CRC. Accumulating studies demonstrated that lncRNAs have a great influence on different biological progresses including apoptosis, cell cycle arrest, EMT, proliferation, invasion and migration. Except that, angiogenesis is essential for tumor growth and an important factor in the metastatic pathway [156, 157]. Accumulating lncRNAs were suggested to promote angiogenesis in liver cancer, non-small cell lung cancer, hepatocellular carcinoma and glioblastoma. However, the association between angiogenesis and lncRNAs in CRC are still not reported yet. Recently, more evidences indicated that angiogenesis may not be necessary for carcinoma metastases [158, 159], whereas co-opting host vessels is the essential choice for tumor cells survive [160]. Surprisingly, vessel co-option may respond for approximately 40% metastases in mCRC [161]. Moreover, evidences showed that the RAS/RAF/MAPK pathway and PI3K/AKT pathway suppresses invasion and promotes EMT, respectively [162, 163]. Therefore, the activation of these pathways might be associated with the phenotype of metastasis in CRC.

Recent studies have demonstrated that genomic variations in lncRNAs, especially in microRNA response elements (MREs) contributed to the risk and progress of CRC [164, 165]. These SNPs may also be associated with CRC metastasis. Because, they may lead to either complete or partial loss of miRNA mediated lncRNAs degradation, resulting in increased lncRNA expression. Moreover, lncRNAs in plasma were also proved to serve as prognostic factor [38, 166]. However, there is no report about the different expression of lncRNAs between early stage CRC patients and mCRC patients. These studies have shed lights on the future research direction on lncRNAs in CRC metastasis. In conclusion, current studies suggest the vital role of lncRNAs in the process of CRC metastasis. More studies with a large cohort of metastatic CRC patients should be performed in the future.
